# HFSUNet: A UNet architecture optimized for precise segmentation of pneumonia lesion boundaries

**DOI:** 10.1016/j.isci.2026.115364

**Published:** 2026-03-13

**Authors:** Zhikun Zhang, Yuntian Zhao, Xiaoping Wu, Zhaoli Yao, Liangquan Jia, Chong Yao, Feng Hua

**Affiliations:** 1 School of Information Engineering, Huzhou University, Huzhou 313000, China; 2 Huzhou Central Hospital, The Affiliated Central Hospital of Huzhou University, No. 1558 Sanhuan North Road, Huzhou 313000, Zhejiang, China

**Keywords:** health sciences, medicine, health informatics, respiratory medicine

## Abstract

Pulmonary lesion segmentation, a key component of computer-aided diagnosis, has significant clinical value in accurately localizing infected regions, quantifying disease progression, and guiding individualized treatment plans. To address the limitations of traditional methods—reliance on handcrafted features, vulnerability to medical image noise, and susceptibility to physician subjectivity—this study proposes HFSUNet, an improved UNet-based segmentation model. The model introduces the multi-scale attention module (MSAM) module to capture cross-scale salient lesion features (alleviating boundary blurring and detail loss), Haar wavelet downsampling (HWD) to reduce downsampling information loss, and marginal weight loss (MWL) to optimize edge segmentation affected by blurred lesion boundaries. Experimental results show HFSUNet achieves mIoU scores of 99.75% (COVID-19), 86.91% (MosMedData), and 92.77%, 92.28%, and 94.16% on DDTI, BUSI, and TN3K, outperforming UNet-based baselines. Notably, while maintaining high segmentation accuracy, it only has 5.638 million parameters and 11.582 GFLOPs, significantly reducing complexity and computational cost. This advantage enables clinical application in resource-limited settings, demonstrating great potential in lesion identification, diagnostic assistance, and prognosis support.

## Highlights

• HFSUNet enhances pneumonia segmentation accuracy and efficiency in resource-limited clinics
• MSAM strengthens lesion feature extraction and solves insufficient feature representation
• HWD and MWL reduce down-sampling loss and improve lesion boundary feature extraction
• The proposed HFSUNet achieves promising performance on multiple medical datasets

## Graphical abstract

**Figure:**
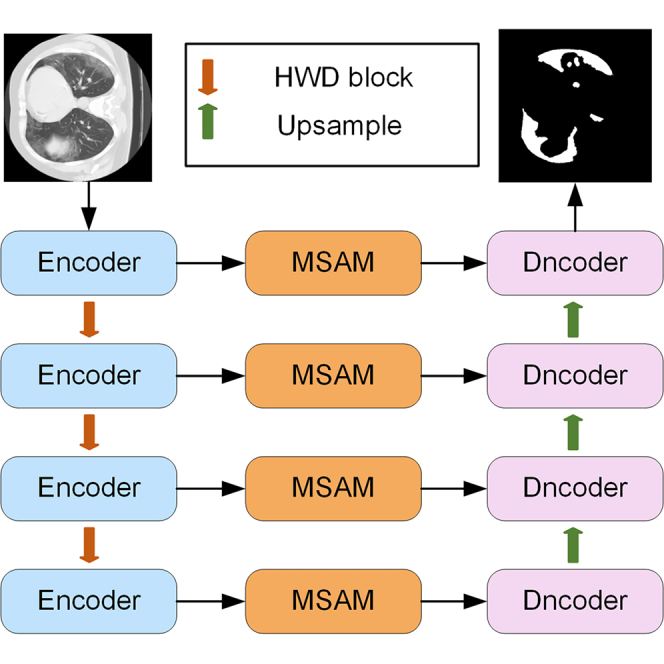

## Introduction

Chest computed tomography (CT) is currently the most widely used and essential imaging technique for the clinical diagnosis of pneumonia, as it can directly reveal the morphology and distribution of lesions. However, the interpretation of CT scans relies heavily on physicians’ professional knowledge and clinical experience.^1^ In practice, manual reading presents three major limitations: first, insufficient diagnostic accuracy, which often leads to missed or incorrect diagnoses^2^; second, the process is time-consuming, making it difficult to meet the high-efficiency demands of clinical settings^3^; and third, the results are easily influenced by subjective judgment, resulting in insufficient consistency.^4^ The root of these challenges lies in the fact that certain non-infectious diseases may also exhibit exudative imaging features similar to pneumonia on CT scans,^5^ thereby increasing the difficulty of interpretation. Consequently, there is an urgent need to develop automated and accurate CT image analysis methods for pneumonia lesion segmentation, in order to enhance the objectivity and reliability of clinical diagnosis and to promote a deeper understanding of the imaging characteristics of pneumonia.

Traditional methods for pneumonia lesion segmentation mostly rely on image processing techniques such as thresholding,^6^ edge detection,^7^ region growing,^8^ and morphological operations. These approaches segment images by extracting grayscale information, texture features, or edge details. Mahaboob et al.^9^ proposed a semi-automatic thresholding method to generate accurate regions of interest (ROI) from lung CT images of COVID-19 positive patients, and further evaluated disease severity by calculating the precise percentage of lung parenchyma involvement (PLA), achieving a specificity of 98.40%. Wang et al.^10^ proposed a noise-robust framework integrated with a noise-resistant Dice loss function for the automatic segmentation of pneumonia lesions from COVID-19 patients’ CT images. However, these conventional methods suffer from several notable limitations in pneumonia lesion segmentation. Firstly, they have high requirements for image quality and are susceptible to noise, artifacts, and other interference, especially exhibiting poor performance in cases of complex lesions and atypical imaging manifestations.^8^ Second, most of these methods rely on handcrafted feature extraction and lack the ability to capture deeper-level image representations, resulting in low accuracy in delineating complex lesions, boundaries, and subtle pathological regions.^11^ Furthermore, traditional methods exhibit poor adaptability, often failing to address inter-patient variability, while also suffering from low processing efficiency.^12^^,^^13^

In recent years, deep learning technologies have achieved breakthrough advancements in the field of medical image processing, successfully addressing bottlenecks of traditional methods such as inefficient manual feature extraction and strong subjectivity.^14^^,^^15^ Among these, the UNet^16^ model stands as a milestone: its revolutionary encoder-decoder architecture enables dual optimization of feature extraction and pixel-level localization. By introducing skip connection mechanisms, UNet not only fully preserves image detail features but also constructs a multi-level semantic association network, thereby simultaneously achieving high-precision reconstruction of target boundaries, exponential optimization of gradient propagation efficiency, and enhanced dynamic stability during training—delivering 92.03% IoU on the PhC-U373 dataset and 77.56% precision on the DIC-HeLa dataset. Since the advent of UNet, researchers have conducted various improvements to further enhance its performance and expand its application scope: U-Net++^17^ innovatively adopts a densely connected nested structure and adaptive feature fusion design to more fully utilize feature information, improve generalization ability, and alleviate the gradient vanishing problem, achieving 92.63% IoU on the Cell nuclei dataset; UNet3+^18^ further leverages the advantages of full-scale skip connections, organically integrating low-level details and high-level semantics from multi-scale feature maps to attain 96.01% Dice coefficient on the ISBI LiTS 2017 Challenge dataset; the Haar wavelet-based downsampling module retains key information while reducing data volume through a unique mechanism, providing an efficient and reliable downsampling solution for semantic segmentation—an UNet incorporating the Haar wavelet downsampling (HWD) module achieved 65.62% performance on the Camvid dataset^19^; the LiteNeXt model combines convolutional and hybrid modules, adopts a simplified decoder structure, and introduces edge weight loss to enhance segmentation boundaries, achieving 92.50% Dice similarity coefficient and 86.39% IoU in the Data Science Bowl 2018.^20^

Despite the significant progress achieved by existing UNet models in pneumonia lesion segmentation, these methods still exhibit notable limitations, particularly regarding the loss of detailed information, difficulties in extracting lesion edge features, and insufficient feature extraction.^8^^,^^17^ Although deep learning models can automatically extract lesion features, in complex imaging scenarios, they fail to fully capture all relevant lesion characteristics—especially when pneumonia lesions are morphologically similar to other lung diseases. The models' ability to identify subtle features remains limited, thereby compromising the discriminative power between pneumonia and other pathological conditions.

**Figure 1:**
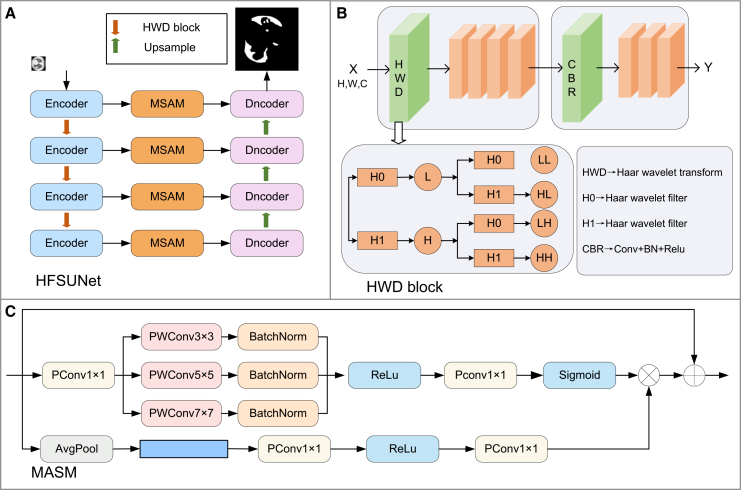
Overall architecture of the model (A) HFSUNet. (B) HWD block. (C) MASM.

**Figure 2:**
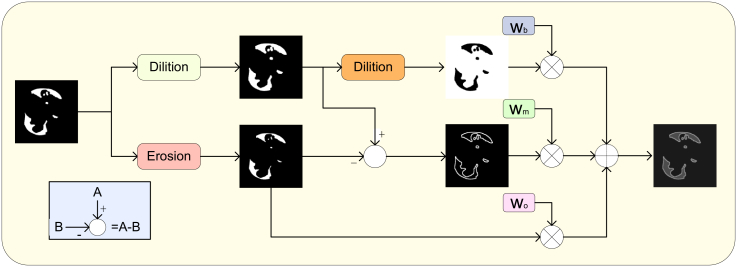
Weight calculation of loss function

To address the technical challenge of balancing efficiency and accuracy in pneumonia image segmentation tasks, this study systematically optimizes UNet as the baseline model. To enhance detection efficiency, a multi-scale attention module (MSAM, see Figure 1B) is innovatively designed to improve the model’s adaptability to lesions of varying sizes; HWD is introduced to replace traditional pooling operations, reducing data dimensionality while preserving lesion details to the greatest extent possible; and marginal weight loss (MWL, see Figure 2) is incorporated to strengthen the model’s ability to learn features from boundary regions. Experimental results demonstrate that this optimization strategy significantly improves both segmentation accuracy and computational efficiency. The main contributions of this study include:

(1) An innovative HFSUNet model for pneumonia segmentation is proposed, which optimizes the network architecture to achieve a synergistic improvement in both detection accuracy and computational efficiency, providing a practical solution for resource-constrained clinical environments.
(2) The design of the MSAM module, based on a multi-scale attention mechanism, significantly enhances lesion feature extraction capability and effectively addresses the problem of insufficient feature representation.
(3) The integration of the HWD module and MWL resolves the challenges of feature loss during down-sampling and the difficulty of extracting features from lesion boundary regions.

## Results

### Dataset

#### COVID-19

This dataset contains chest CT scans from 20 clinically confirmed patients with COVID-19.^21^ All imaging data were processed with standardized lung window settings, and the left lung, right lung, and infected regions were independently annotated by two experienced thoracic radiologists. The annotations were further validated by a senior expert in chest imaging diagnostics to ensure accuracy, meeting clinical diagnostic standards.

#### MosMedData

Collected by a medical institution in Moscow, Russia, between March 1 and April 25, 2020, the MosMedData dataset^22^ comprises 1,000 anonymized human lung CT images, all sourced from non-duplicate independent patients. Among these, 50 studies have been professionally annotated to generate binary pixel-level masks for the accurate segmentation of lung ROIs, covering typical COVID-19 imaging features such as ground-glass opacity (GGO) and lung tissue consolidation. In this study, these 50 professionally annotated cases were utilized to conduct generalization research on pneumonia segmentation.

#### DDTI

The DDTI dataset,^23^ provided by Pedraza et al., consists of 637 thyroid ultrasound images acquired from a single device, each with fine-grained pixel-level annotations. Its value lies in covering a wide range of pathological conditions, including thyroiditis, goiter, nodules, and cancer, thereby reflecting the complexity and challenges of thyroid ultrasound image analysis. Since thyroid nodules are often irregular in shape, have blurred boundaries, and present complex echo characteristics that can easily be confused with surrounding normal tissue, their detection and segmentation remain highly challenging. With its diagnostic diversity and high image complexity, the DDTI dataset provides critical support for clinical diagnosis and prognosis assessment. In particular, for the task of thyroid nodule segmentation, the dataset’s broad pathological coverage and detailed annotations establish a solid foundation for tackling issues such as boundary ambiguity and feature overlap, and for developing more accurate segmentation strategies.

#### BUSI

The BUSI^24^ dataset is a breast ultrasound image collection for both classification and segmentation tasks. It includes ultrasound images of 600 female patients aged 25–75, collected in 2018. Comprising 780 images with an average size of 500 × 500 pixels, the dataset is categorized into three classes: normal, benign, and malignant. For both benign and malignant cases, detailed segmentation annotations of corresponding breast tumors are provided, offering critical information for in-depth research and precise diagnosis.

#### TN3K

The TN3K^25^ thyroid nodule segmentation dataset is a high-quality medical imaging benchmark specifically developed for computer-aided diagnosis of thyroid nodules. It contains 3,493 ultrasound images from 2,421 patients (PNG format), collected between January 2016 and August 2020. These images were rigorously selected from over 30,000 raw images provided by collaborating hospitals—systematically choosing only those containing at least one clearly defined thyroid nodule without significant lymphatic tissue interference or large-scale color artifacts. A view-region dual deduplication strategy was employed to retain the most diagnostically representative images. After review by senior physicians from tertiary hospitals, the dataset was divided into 2,879 training images and 614 independent test images. This dataset achieves industry-leading standards in sample diversity, annotation quality, and clinical practicality. Its thyroid nodule ultrasound segmentation task holds significant clinical value for early thyroid cancer diagnosis, providing a reliable benchmark for deep learning model development and supporting cross-institutional collaborative research.

### Comparative study

To evaluate the performance of the HFSUNet model, we conducted comprehensive training on the COVID-19 dataset and performed a systematic comparative analysis against baseline UNet architectures. The comparative study incorporated seven representative UNet variants, including UNet, UNet++, UNet3+, CMUNet,^26^ AttUNet,^27^ UNext,^28^ and Cmunext.^29^ In the evaluation process, we not only focused on the detection performance but also conducted a quantitative comparison of the number of parameters, floating-point operations per second (GFLOPs), and the average inference time (in milliseconds) required for processing a single CT slice across different models. Detailed comparison data are presented in Table 1.

**Table 1:** Model performance metrics on the COVID-19 dataset

Model	MIoU (%)	Dice (%)	F1_score	Param (M)	GFLOPs	Time
UNet	99.70	99.85	99.85	34.526	65.447	38.76
AttUnet	99.65	99.82	99.82	34.877	66.556	38.61
UNet++	99.69	99.84	99.84	26.898	37.523	36.42
UNet3+	99.72	99.86	99.86	26.97	199.667	51.97
CMUNet	99.68	99.84	99.84	34.218	69.371	39.19
UNext	99.56	99.78	99.78	1.471	0.554	25.28
Cmunext	99.63	99.82	99.82	3.149	7.399	28.37
HFSUNet	99.75	99.87	99.87	5.638	11.582	29.84

**Figure 3:**
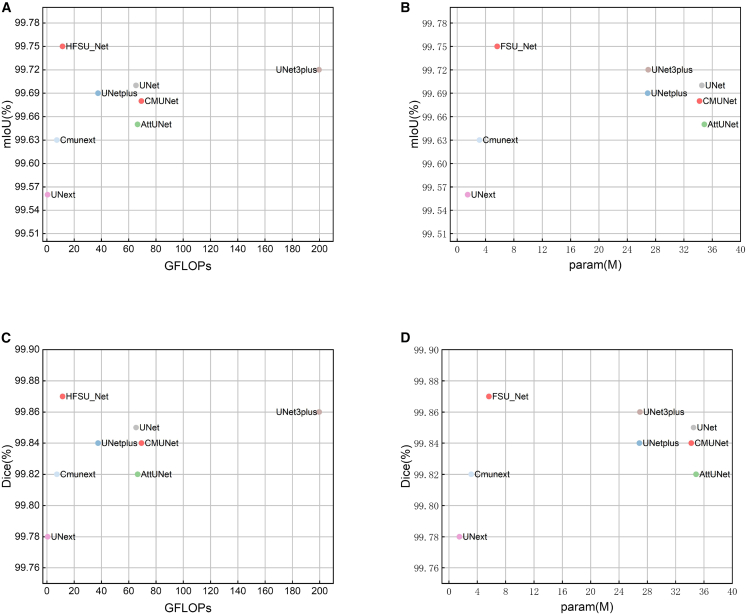
Performance-efficiency comparison of HFSUNet and baseline models on the COVID-19 (A) Scatterplot of mIoU vs. GFLOPs. (B) Scatterplot of mIoU vs. parameter count. (C) Scatterplot of Dice vs. GFLOPs. (D) Scatterplot of Dice vs. parameter count.

From the quantitative analysis results, HFSUNet demonstrates significant advantages in segmentation performance, with all evaluation metrics surpassing those of the comparison models, thereby fully validating the effectiveness of the proposed method. To further investigate the balance between performance and efficiency, this study plotted scatter diagrams of mIoU and Dice (performance dimensions) against GFLOPs and number of parameters (efficiency dimensions) for HFSUNet and the baseline models on the COVID-19 (Figure 3), illustrating the distribution characteristics of different models in the performance-efficiency space. The results show that HFSUNet not only maintains excellent segmentation accuracy but also achieves a substantial reduction in computational resource consumption, which highlights the innovativeness of its network architecture design and its outstanding capability in balancing performance and efficiency.

To systematically and comprehensively evaluate the performance of the HFSUNet model in pneumonia lesion segmentation, this study further conducted detailed comparative experiments on the publicly available MosMedData medical imaging dataset. In these experiments, we performed a meticulous performance comparison between the proposed HFSUNet model and current mainstream baseline models, with the relevant quantitative results presented in Table 2.

**Table 2:** Performance comparison of models on the MosMedData dataset

Model	MIoU (%)	Dice (%)	F1_score
UNet	85.9	92.15	86.79
AttUnet	87.53	93.14	88.45
UNet++	84.26	90.96	83.64
UNet3+	86.23	92.35	87.1
CMUNet	87.07	92.89	87.91
UNext	73.26	83.5	72.33
Cmunext	62.86	73.39	51.54
HFSUNet	86.91	92.76	87.75

HFSUNet demonstrates an outstanding precision-efficiency balance in pneumonia segmentation tasks: its mIoU, Dice, and F1 scores reach 86.91%, 92.76%, and 87.75%, respectively, with minimal gaps of only 0.62%, 0.38%, and 0.70% compared to the current state-of-the-art AttUnet model—proving its segmentation accuracy has achieved a high level. Meanwhile, HFSUNet features a computational complexity of merely 5.638M parameters and 11.582 GFLOPs, significantly lower than high-precision models such as UNet, AttUnet, and CMUNet, which substantially reduces memory usage and inference latency. Compared to the lightweight UNext model, although HFSUNet has a slight increase in parameters and computational load, its Dice and F1 scores are drastically improved by 9.26% and 15.42% respectively, achieving the optimization goal of “fewer resource inputs and higher segmentation efficiency.”

To systematically evaluate the cross-dataset generalization capability of HFSUNet, three clinically representative medical imaging datasets—DDTI, BUSI, and TN3K—were selected for testing. In the experiments, HFSUNet was compared with multiple baseline models (results shown in Table 2) to comprehensively validate its applicability and robustness across diverse clinical scenarios.

**Figure 4:**
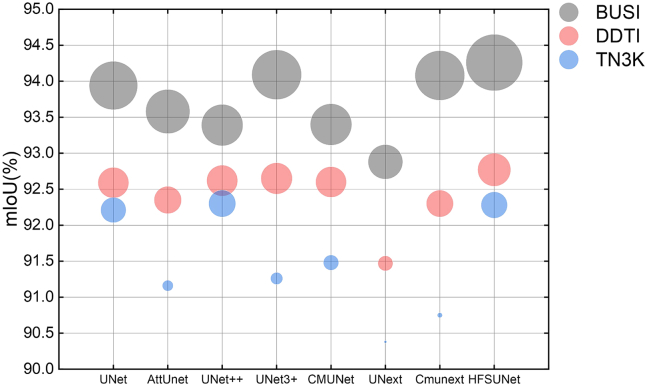
mIoU comparison bubble charts of different models on three public datasets

To more clearly illustrate the performance of HFSUNet across different medical imaging datasets, this study plotted mIoU bubble charts on the DDTI, TN3K, and BUSI datasets, comparing HFSUNet with UNet, UNet++, UNet3+, CMUNet, AttUNet, UNext, and CMUNext, as shown in Figure 4.

Based on the parameter and FLOPs statistics in Table 2, together with the mIoU comparison results of HFSUNet and baseline models on the DDTI, TN3K, and BUSI medical datasets shown in Table 3 and Figure 4, it can be concluded that HFSUNet not only achieves substantially lower computational cost than other state-of-the-art models but also surpasses them in segmentation accuracy, demonstrating excellent generalization performance.

**Table 3:** Comparison of mIoU (%) data on DDTI, TN3K, and BUSI datasets

Model	DDTI	TN3K	BUSI
UNet	92.59	93.94	92.21
AttUnet	92.35	93.58	91.16
UNet++	92.62	93.39	92.30
UNet3+	92.65	94.09	91.26
CMUNet	92.60	93.40	91.48
UNext	91.47	92.88	90.38
Cmunext	92.30	94.08	90.75
HFSUNet	92.77	94.16	92.28

**Figure 5:**
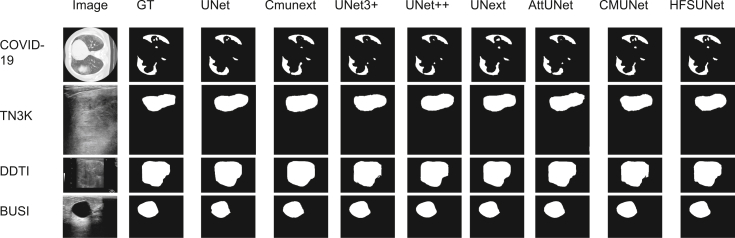
Visualization results

Comprehensive experimental results across three publicly available datasets—DDTI, BUSI, and TN3K—demonstrate that HFSUNet consistently achieves significant segmentation performance improvements. To visually compare the segmentation capabilities of different models across four datasets (including COVID-19), we present systematic visualization results in Figure 5. As illustrated, HFSUNet exhibits markedly superior lesion boundary delineation capabilities, particularly in complex edge regions, where it demonstrates exceptional precision in segmenting intricate boundaries.

### Ablation study

To systematically evaluate the performance advantages of the MWL, we designed and conducted a series of rigorous ablation experiments. By strictly controlling experimental variables, we maintained consistent configurations across all factors except the loss function—including the HFSUNet architecture, training hyperparameters, and data preprocessing protocols. Four authoritative medical imaging datasets—COVID-19, DDTI, BUSI, and TN3K—were selected as testing platforms to systematically compare the segmentation performance of MWL against cross-entropy loss, Dice loss, weighted binary cross-entropy loss (W-BCE),^30^ and local boundary-balanced loss (LB-BCE).^31^ The experimental results presented in Table 4 demonstrate that MWL achieves statistically significant performance improvements across all evaluated datasets, validating its effectiveness for medical image segmentation tasks.

**Table 4:** Experimental results of loss function ablation

Loss	COVID-19	DDTI	TN3K	BUSI
CE Loss	99.67	92.34	93.54	92.14
Dice Loss	99.65	92.59	93.28	92.17
W-BCE	99.69	92.73	94.11	92.13
B-BCE	99.74	92.68	94.07	92.01
MWL	99.75	92.77	94.16	92.28

**Figure 6:**
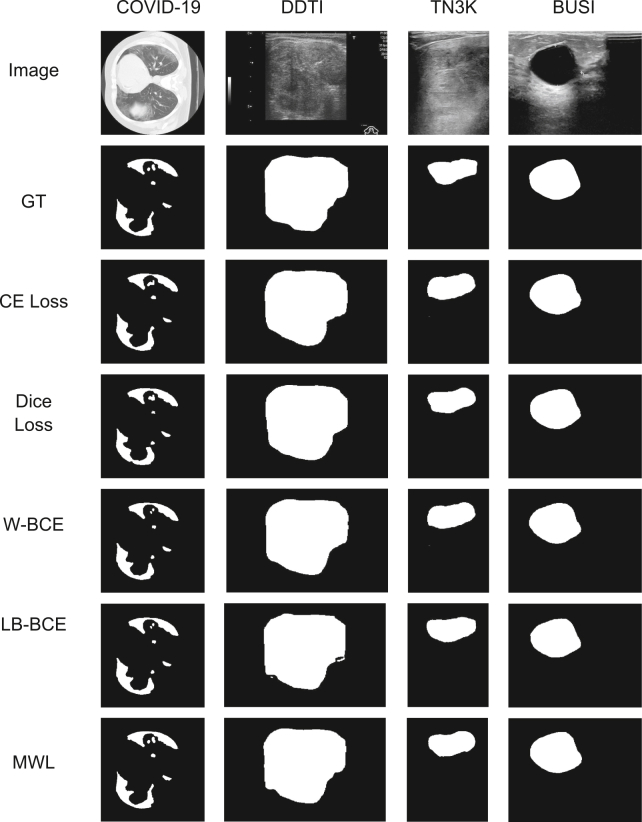
Comparison of loss functions

To validate the performance advantages of the MWL in enhancing edge information extraction, we employed a controlled variable approach. Using identical HFSUNet architectures, we trained models with five distinct loss functions—MWL, cross-entropy loss, Dice loss, W-BCE, and LB-BCE—and conducted systematic visual comparisons of segmentation results. As qualitatively demonstrated in Figure 6, the model trained with MWL exhibits remarkable superiority in edge information extraction: It not only preserves the structural integrity of lesion regions but also precisely localizes fine-grained boundary structures in high-resolution images. Particularly in tasks involving thin-structure contour extraction and complex edge transition segmentation, MWL demonstrates superior recognition accuracy. Crucially, compared to conventional loss functions, the MWL-trained model effectively mitigates common issues such as edge blurring and over-segmentation, achieving optimal edge preservation while maintaining high segmentation accuracy. These findings not only verify the innovation and practical value of the MWL module in edge extraction but also provide robust technical support for clinical applications—such as medical image analysis—where boundary localization precision is critically required. Furthermore, this work establishes a solid theoretical foundation for exploring future model optimization pathways and expanding application domains.

To systematically evaluate the performance advantages of the HWD Module in medical image segmentation tasks, we designed and conducted a series of multi-dataset comparative experiments. The HWD module was comprehensively evaluated against traditional max pooling and average pooling methods across four authoritative medical imaging datasets: COVID-19, DDTI, BUSI, and TN3K. As clearly demonstrated in Table 5, the HWD module achieves consistent and significant segmentation performance improvements across all evaluated datasets: specifically, mIoU enhancements of 0.03% on COVID-19, 0.09% on DDTI, 0.09% on TN3K, and 0.12% on BUSI, all showing superior performance compared to max pooling.

**Table 5:** HWD module ablation experiment results

	COVID-19	DDTI	TN3K	BUSI
MaxPooling	99.72	92.68	94.07	92.26
AvgPooling	99.68	92.24	93.87	92.13
HWD	99.75	92.77	94.16	92.28

### Paired t-test

**Figure 7:**
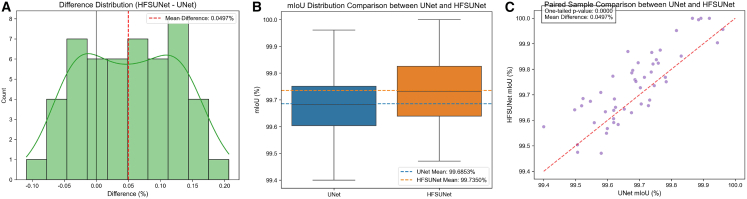
Sample *t* test analysis chart (A) Difference distribution (HFSUNet-UNet). (B) mIoU Distribution comparison between UNet and HFSUNet. (C) Paired sample comparison between UNet and HFSUNet.

To verify the reliability of HFSUNet’s performance improvement over UNet, statistical analysis and visualization validation were conducted on the mIoU data of 50 paired test samples (Table 6; Figure 7). The statistical results show that the mean mIoU of HFSUNet is 99.7350%, which is 0.0497% higher than UNet’s 99.6853%. The median difference is 0.0530%, which is nearly consistent with the mean difference, indicating that the improvement represents a stable overall trend without interference from extreme values (difference range: −0.1091%–0.2067%, SD = 0.0766%). The paired *t* test results reveal t = 4.5876 (df = 49) and a one-tailed *p*-value of 1.6 × 10^−5^ (*p* < 0.001). Combined with a medium effect size (Cohen’s d = 0.6488), these findings confirm that the performance difference is statistically extremely significant and of practical application value.

**Table 6:** Statistical comparison of mIoU performance between UNet and HFSUNet

Statistical Metrics	UNet (%)	HFSUNet (%)	Difference (%)
Mean	99.6853	99.735%	0.0497
Median	99.6821	99.732	0.053
SD	0.1238	0.1402	0.0766
Min	99.4	99.471	−0.1091
Max	99.961	100	0.2067
SE	0.0175	0.0198	0.0108
Paired t-test statistic	–	–	t = 4.5876, df = 49
One-tailed *p*-value	–	–	1.6 × 10^−5^
Effect size	–	–	0.6488

The visualization results further confirm the reliability: The boxplot (Figure 7A) shows that the performance distribution of HFSUNet is overall shifted upward, forming a clear separation from UNet; the difference distribution plot (Figure 7B) is concentrated around positive values, with the mean difference line far from the zero line, verifying the consistency of the improvement; in the paired scatterplot (Figure 7C), 64% (32 out of 50 groups) of the sample points lie above the y = x reference line, and among the samples with significant differences (absolute difference >0.1%), 94.4% (17 out of 18 groups) exhibit positive differences—highlighting the dominant advantage of HFSUNet in most scenarios, especially complex segmentation tasks. In summary, the statistical data and visualization results mutually corroborate, indicating that the mIoU performance improvement of HFSUNet possesses both statistical rigor and practical stability.

### Qualitative analysis of challenging samples

**Figure 8:**
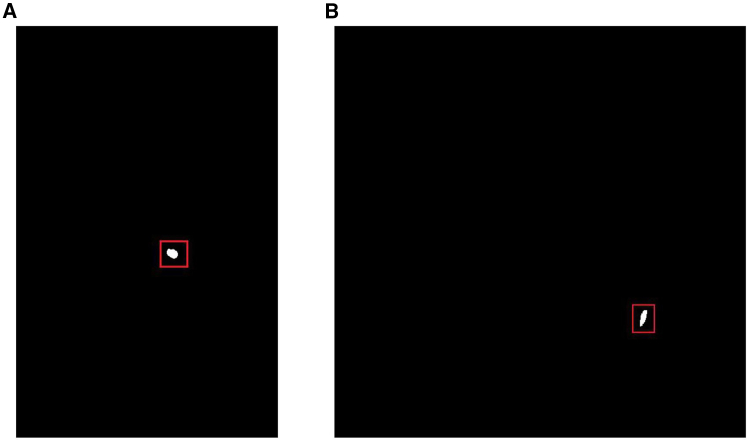
Example of small lesions CT (A) Segmentation of a small. (B) Segmentation of an elongated.

Through systematic analysis of test set data, this study reveals significant limitations of current models in segmenting small lesions. The test set contains a large number of small lesion samples (as shown in Figure 8), with some cases presenting only small lesions (single lesion pixel area <100 pixels)—these lesions are typically challenging for segmentation tasks. Statistical analysis shows that among 72 small lesions with a single lesion pixel area of less than 100 pixels, the model successfully segmented 53, but with suboptimal performance, while as many as 19 lesions (26.4%) were completely undetected. Notably, although these small lesions account for only a minimal proportion of the test set, they exert a disproportionate impact on overall performance metrics: In 10 CT images containing only a single undetected small lesion, the model’s overall evaluation metric (mIoU) still reached as high as 90%. This phenomenon highlights the limitations of conventional evaluation metrics in reflecting a model’s ability to handle small lesions.

By delving into the mismatch between inherent model architectural characteristics and the intrinsic features of small lesions, this study summarizes four key failure factors: First, the multi-layer downsampling design inherent in standard UNet architectures gradually compresses feature map sizes from the original resolution. Small lesions (<100 pixels) are often compressed to 1–2 pixels or even completely disappear during this process, resulting in the total loss of positional and morphological information of these critical lesions in high-level feature representations. Second, the HU value difference between small lesions and surrounding lung tissue is extremely subtle (ΔHU <50), and this difference is further smoothed after standardization preprocessing, making it difficult for models to effectively extract lesion-specific edge or texture features using conventional 3 × 3 convolution kernels. Third, the limited receptive field of shallow convolution kernels in standard UNet fails to fully capture the weak contrast differences between small lesions and surrounding lung textures, while lacking an effective mechanism for integrating global contextual information. Finally, the pixel-wise equal weighting design of the cross-entropy loss function causes the gradient contributions of small lesions to be “drowned out” by the dominant large background regions. This makes the model optimization process naturally inclined to prioritize improving segmentation accuracy for large lesions or background regions, while neglecting the detailed processing of small targets.

The results of this study clearly demonstrate the significant inadequacy of current models in segmenting small lesions, which constitutes a critical bottleneck limiting overall model performance. This finding holds important implications for the practical clinical application of models: Although models perform excellently in handling lesions of common sizes, their ability to detect early-stage or small lesions is significantly limited. This may lead to the omission of key diagnostic information in clinical practice, thereby affecting the early detection of diseases and treatment decision-making. To address this limitation, future research should focus on developing strategies to enhance model sensitivity to small targets. Potential solutions include: introducing multi-scale feature fusion mechanisms to expand receptive field coverage for small targets; designing shallow high-resolution preservation branches to maintain fine-grained spatial information; developing loss functions optimized for small targets to balance optimization weights among lesions of different sizes; or integrating adaptive post-processing techniques to improve segmentation accuracy of small connected regions. Exploration of these technical directions will help compensate for the shortcomings of current models in small lesion segmentation and enhance their practical value and reliability in clinical practice.

## Discussion

This study proposes HFSUNet, a lightweight UNet variant for pneumonia lesion segmentation, specifically optimized to address the limitations of insufficient boundary detail extraction and segmentation accuracy. The model integrates three key components: MASM, which enhances cross-scale information interaction and balances the capture of fine-grained structures with high-level semantic features; HWD, which reduces computational complexity while minimizing information loss, showing particular advantages in edge preservation and small lesion segmentation; and MWL, which dynamically adjusts loss weights to significantly improve segmentation accuracy in complex boundary regions.

In terms of performance, HFSUNet achieved 99.75% mIoU and 99.87% Dice coefficient on the COVID-19, outperforming traditional UNet and its variants, while substantially reducing the number of parameters (5.638M) and computational cost (11.582 GFLOPs), thus demonstrating efficiency and practicality. Ablation studies further confirmed the independent contributions of each module: MASM significantly enhanced multi-scale feature fusion, HWD achieved the best performance in edge detail preservation, and MWL consistently outperformed conventional loss functions across multiple datasets. Cross-dataset evaluations on DDTI, BUSI, and TN3K further validated the generalization capability of HFSUNet and its adaptability to diverse clinical scenarios.

### Limitations of the study

Despite these promising results, several limitations remain. First, model training and validation primarily relied on COVID-19-related imaging data, leaving its performance on other types of pneumonia (e.g., bacteria or fungal) and non-infectious pulmonary diseases unverified. Second, COVID-19 pneumonia presents specific imaging characteristics, which may lead to etiology-dependent feature representations. Third, the reliance on a single-disease dataset fails to capture the morphological, density, and distributional variations across different etiologies, potentially overestimating the model’s generalization ability.

## Future expectations

To address these limitations, future work will focus on three directions: (1) constructing large-scale, multi-center, and cross-regional pneumonia CT databases that include diverse geographical, medical, and demographic characteristics—especially vulnerable groups such as children and the elderly—while implementing unified annotation protocols and quality-control procedures to minimize subjectivity; (2) incorporating regional information and multi-task learning mechanisms into model design, enabling simultaneous pneumonia segmentation and region-specific feature recognition, thereby improving robustness across regions; and (3) conducting prospective multi-regional clinical trials to continuously optimize model performance and validate its clinical feasibility, ultimately promoting the application of HFSUNet in primary healthcare institutions and supporting intelligent pneumonia diagnosis in resource-constrained environments.

## Resource availability

### Lead contact

For further information or to request resources and reagent-related materials, please contact the lead contact Liangquan Jia, who will coordinate the fulfillment of relevant requests. Contact information: 02426@zjhu.edu.cn.

### Materials availability

This study did not generate new unique reagents.

### Data and code availability

• The source code used in this study is not yet publicly available and can be obtained by contacting the lead contact.
• The datasets analyzed in this paper (COVID-19, MosMedData, DDTI, BUSI, and TN3K) are existing publicly available datasets, and the relevant literature can be found in the reference list.
• Any additional information required to reanalyze the data reported in this paper is available from the lead contact upon request.

## Acknowledgments

This research was supported by the Huzhou Public Welfare Applied Research Project (Key Project) [2023GZ84], the Huzhou Public Welfare Applied Research Project (General Project) [2021GY35], and the Postgraduate Research and Innovation Project of 10.13039/100012844Huzhou University [2025KYCX66].

## Author contributions

Z.Z. and Y.Z. conceived and conducted the experiments and drafted the original manuscript. X.W. and Z.Y. supervised the study. L.J., C.Y., and F.H. contributed to the formal analysis. All authors reviewed and approved the final text.

## Declaration of interests

The authors declare no competing interests.

## STAR★Methods

### Key resources table

**Table:** 

REAGENT or RESOURCE	SOURCE	IDENTIFIER
**Software and algorithms**		

Python	Python Software Foundation	3.8 / SCR_008394; https://www.python.org/
Pytorch	Facebook	2.3.0 / SCR_018536; https://pytorch.org/
opencv	Opencv library	4.8.0 /SCR_015526; https://opencv.org/
numpy	Array programming with NumPy	1.26 / SCR_008633; https://numpy.org/
Scikit-learn	Machine learning in Python	1.3.0 / SCR_002577; https://scikit-learn.org/stable/index.html

### Experimental model and study participant details

In this study, we conducted extensive and in-depth experiments to systematically evaluate the performance of the proposed HFSUNet model in pneumonia segmentation tasks. Two pneumonia CT datasets (COVID-19 and MosMedData) were selected for core model assessment, with each dataset randomly split into a training set (70%), test set (20%), and validation set (10%). Additionally, three public datasets (DDTI, TN3K, and BUSI) were adopted to verify the model’s cross-scenario generalization capability. All experiments were performed on a computer running the Windows 10 Pro (64-bit) operating system, equipped with an Intel 12th Generation Core i5-12400F processor (base frequency: 2.50 GHz), 32 GB Kingston DDR4 3200 MHz memory (16 GB × 2), and an NVIDIA GeForce RTX 4070 graphics card.

To enhance the model’s robustness and generalization ability, a multi-dimensional data augmentation strategy was employed, including random horizontal flipping, vertical flipping, scaling, random cropping, and contrast adjustment. The training parameters were set as follows: batch size = 8, total training steps = 1000, optimizer = RMSprop, and initial learning rate = 0.0001. This data augmentation strategy achieved three key benefits: first, it effectively mitigated model overfitting by simulating variations in imaging perspectives and natural organ deformations; second, results on the validation set showed that the model’s accuracy improved by approximately 0.12%–0.17%; third, the augmented data distribution increased the model’s inference speed by 3 times, significantly optimizing computational efficiency. Furthermore, an early stopping strategy (with a patience value of 50) was introduced during the training process to further prevent overfitting.

### Method details

#### Overall architecture

The architecture of the proposed HFSUNet is illustrated in Figure 1A. The overall framework consists of four hierarchical levels and can be logically divided into three core components: the encoder module, the multi-scale skip connection module, and the decoder module. In the encoder stage, the HWD module is innovatively introduced, replacing conventional down-sampling methods with the multi-resolution property of wavelet transforms. This design reduces the resolution of feature maps while effectively preserving detailed information, thereby significantly alleviating the feature loss typically encountered in standard down-sampling processes. In addition, a multi-scale feature extraction module is designed as a bridge between the encoder and decoder, enabling the capture of features at different scales and further enhancing the model’s feature representation capability. Moreover, given the critical role of edge details in pneumonia segmentation tasks, a MWL function is incorporated, which optimizes the loss design to specifically improve the model’s sensitivity and accuracy in recognizing lesion boundary features. Through the synergistic effect of these improvements, HFSUNet substantially enhances the performance of the UNet model in pneumonia CT image segmentation, with particularly notable improvements in boundary localization and detail preservation.

#### Haar wavelet downsampling

The Haar wavelet down-sampling (HWD) module is an innovative component that integrates the characteristics of wavelet transform with convolutional neural networks (Figure 1B). Its primary objective is to reduce data dimensionality while effectively preserving critical image features. Built upon the Haar wavelet transform, the module employs low-pass filtering to extract approximation coefficients for retaining the global structure, while simultaneously leveraging high-frequency components (LH, HL, HH) to encode boundary irregularities and internal texture details of lesions. This design addresses the staircase artifacts typically introduced by max pooling and the detail blurring caused by average pooling, making it particularly well-suited for accurate representation of pneumonia lesions with complex morphologies. By further combining convolution, batch normalization, and activation operations, the HWD module demonstrates notable advantages in multi-scale feature extraction: a single decomposition can simultaneously capture multi-frequency representations ranging from lobe-level consolidations to sub-millimeter early exudative lesions, thereby avoiding the feature dilution of small lesions that commonly occurs with multi-layer stacked pooling operations.

The Haar wavelet transform can be decomposed into wavelet transforms along the row and column directions. At each stage, a low-pass filter and a high-pass filter are applied to extract the approximate information (low-frequency components) and detail information (high-frequency components) of the image. The first-stage Haar wavelet transform can be defined as follows:

(Equation 1) $$\begin{array}{c} \left\{ \begin{array}{c} L_{\text{row}}\left(i,j\right)=\frac{I\left(i,2j\right)+I\left(i,2j+1\right)}{\sqrt{2}}\\ H_{\text{row}}\left(i,j\right)=\frac{I\left(i,2j\right)-I\left(i,2j+1\right)}{\sqrt{2}}\; \end{array}\right. \end{array}$$

Here, L_row_ represents the low-frequency components along the row direction, while H_row_ denotes the high-frequency components along the row direction. In the second stage, a one-dimensional Haar transform is applied to each column of L_row_ and H_row_, which can be defined as follows:

(Equation 2) $$\begin{array}{c} \left\{ \begin{array}{c} L_{\text{LL}}\left(m,n\right)=\frac{L_{\text{row}}\left(2m,n\right)+L_{\text{row}}\left(2m+1,n\right)}{\sqrt{2}}\\ L_{\text{LH}}\left(m,n\right)=\frac{L_{\text{row}}\left(2m,n\right)-L_{\text{row}}\left(2m+1,n\right)}{\sqrt{2}}\\ H_{\text{HL}}\left(m,n\right)=\frac{H_{\text{row}}\left(2m,n\right)+H_{\text{row}}\left(2m+1,n\right)}{\sqrt{2}}\\ H_{\text{HH}}\left(m,n\right)=\frac{H_{\text{row}}\left(2m,n\right)-H_{\text{row}}\left(2m+1,n\right)}{\sqrt{2}} \end{array}\right. \end{array}$$

The LL subband, functioning as the low-frequency component, captures the fundamental structural features of the image through an approximation filtering mechanism, primarily characterizing the overall contour distribution and spatial continuity within smooth regions. The LH subband concentrates on high-frequency details in the horizontal direction, with its activation patterns precisely matching abrupt transitions in vertical edges. Meanwhile, the HL subband analyzes high-frequency information in the vertical direction, specifically identifying horizontal edge variations. In turn, the HH subband targets high-frequency components along diagonal orientations, effectively extracting complex texture information featuring 45° or 135° angled edges. This orthogonal directional decomposition mechanism enables joint spatial-frequency representation across multiple scales. The four subbands are concatenated along the channel dimension to construct a multi-scale feature spectrum, which subsequently undergoes cross-component correlation feature extraction via convolutional layers, feature distribution stabilization through batch normalization layers, and non-linear transformation introduction by ReLU activation functions—ultimately producing fusion features with enhanced representational capacity. This approach comprehensively preserves critical structural information during downsampling, demonstrating particular suitability for extracting multi-dimensional features from the complex morphologies of pneumonia lesions.

#### Multi-Scale Attention Module

In pneumonia CT image segmentation, existing methods often suffer from insufficient multi-scale adaptability, loss of lesion boundary details, and inadequate utilization of channel features, resulting in suboptimal performance in complex lesions and atypical cases. To address these shortcomings, this study proposes a Multi-Scale Attention Module (MASM) (Figure 1C), which, when combined with skip connections, suppresses irrelevant features while enhancing valuable ones. The module employs a dual-path collaborative mechanism—multi-scale feature refinement in the spatial dimension and attention-based aggregation in the channel dimension—to dynamically adjust feature weights. Finally, through residual connections, the weighted features are added back to the original input, thereby ensuring information integrity while effectively mitigating the problem of gradient vanishing.

Specifically, MASM consists of two core branches: a spatial refinement branch and a channel aggregation branch. In the spatial branch, pointwise convolution is first applied to transform the input features along the channel dimension, moderately compressing the number of channels to reduce subsequent computational cost while preserving key inter-channel information. Then, three parallel depthwise convolutions are employed to extract multi-scale features, followed by BatchNorm to stabilize training and ReLU activation to introduce nonlinear representation. Afterward, another pointwise convolution is used to adjust the channel dimension, and a Sigmoid activation generates the spatial attention weight map, which precisely characterizes the importance of each spatial position in the feature map. The channel aggregation branch focuses on global context modeling: global average pooling is first applied to compress the spatial information of each channel into a single value, thereby extracting channel-level global features; these are then passed through two successive pointwise convolutions with ReLU activation to generate channel-attention-related features, ultimately forming the channel attention map that represents the importance of each channel.

The outputs of the two branches are further fused by element-wise multiplication of the spatial attention weights with the multi-branch convolutional features, thereby dynamically weighting key spatial positions in the feature map. At the same time, the generated channel attention map is combined with the spatially refined features to achieve channel-wise feature selection. Finally, the weighted features are fused with the original input through residual connections—this design not only preserves the detailed features captured by shallow layers but also incorporates the semantic information learned by deeper layers. Moreover, the direct cross-layer connections alleviate the gradient vanishing problem in deep networks, thereby significantly enhancing the module’s feature representation capacity and training stability.

#### Marginal weight loss

Pneumonia lesions exhibit a series of distinctive characteristics on CT images. Their edges demonstrate significant blurriness, making it difficult to precisely delineate the boundaries between the lesions and surrounding normal tissues. Morphologically, the lesions are highly irregular, lacking fixed geometric shapes, which complicates the segmentation process. Additionally, compared to adjacent normal tissues, the lesions exhibit lower contrast, further increasing the difficulty of segmentation. The combination of these features results in substantially lower segmentation accuracy in the boundary regions of the lesions compared to the interior regions of the lesions and the background areas. Moreover, the background regions in CT images typically occupy the majority of the image area. Traditional loss functions, which fail to adequately account for class imbalance issues, tend to overly focus on the dominant class (i.e., the background) during loss computation. Consequently, the optimization effect on lesion regions—particularly the boundary regions—is severely weakened. This imbalanced treatment ultimately significantly degrades the model’s overall segmentation performance for pneumonia lesions, rendering the segmentation results insufficient to meet the precision requirements of medical diagnosis.

To effectively address the aforementioned challenges, this study introduces MWL, whose core concept is to implement differentiated treatment of different regions through a dynamic weight allocation mechanism (Figure 2). The key innovation lies in establishing an adaptive weighting scheme based on segmentation difficulty. In pneumonia lesion segmentation, regional difficulty varies significantly: lesion boundaries, due to their blurriness, irregularity, and low contrast, are considered high-difficulty areas where segmentation accuracy is typically low. These regions are therefore assigned higher weights to guide the model in strengthening feature learning and improving boundary precision. In contrast, easily segmentable regions such as the background are assigned lower weights to avoid model overfitting, allowing more learning resources to be concentrated on challenging regions. Compared with traditional loss functions, MWL enables fine-grained control of pixel-level weights, which not only maintains stable convergence but also significantly enhances segmentation accuracy for pneumonia lesion boundaries. The mathematical formulation of MWL is given as follows:

(Equation 3) $$\begin{array}{c} W_{e}=Erosion\left(GT\right) \end{array}$$

(Equation 4) $$\begin{array}{c} W_{d}=Dilation\left(GT\right) \end{array}$$

(Equation 5) $$\begin{array}{c} {\text{Weight}}_{mask}=\left(w_{b}\cdot \text{Inversion}\left(W_{d}\right)+w_{m}\cdot \left(W_{d}-W_{e}\right)+w_{0}\cdot W_{e}\right) \end{array}$$

(Equation 6) $$\begin{array}{c} L_{MWL}\left({\hat{y}}_{i},y_{i}\right)=-\sum_{i=1}^{H\times W}\;{\text{Weight}}_{mask}\left(i\right)\left(y_{i}\;\log\left({\hat{y}}_{i}\right)+\left(1-y_{i}\right)\log\left(1-{\hat{y}}_{i}\right)\right) \end{array}$$

Here, GT denotes the ground truth labels, Erosion represents the erosion operation, Dilation refers to the dilation operation, and Inversion indicates the inversion operation. The parameters w_b_, w_m_, and w_0_ are hyperparameters designed to assign weights to different target regions, while Weight_mask_ is the masking matrix. $L_{\text{MWL}}\left({\hat{y}}_{i},y_{i}\right)$ represents the final computed loss value.

### Quantification and statistical analysis

#### Evaluation metrics

In scientific research and data analysis, classification model performance is commonly evaluated using four fundamental metrics: True Positive (TP) refers to cases where both the actual and predicted values are positive, meaning the model correctly identifies an event as occurring; True Negative (TN) describes scenarios where both actual and predicted values are negative, indicating the model accurately recognizes an event as not occurring; False Positive (FP) occurs when the actual value is negative but the model incorrectly predicts a positive outcome, representing a Type I error; False Negative (FN) arises when the actual value is positive but the model erroneously predicts a negative result, constituting a Type II error.

The mean Intersection over Union (mIoU) is a widely used metric for evaluating the performance of semantic segmentation models. It is calculated by averaging the Intersection over Union (IoU) values across all classes. IoU, a fundamental evaluation metric in object detection and semantic segmentation, measures the overlap degree between model-predicted regions (either bounding boxes or pixel-level predictions) and ground truth targets, serving as a critical indicator of model accuracy. The mIoU calculation involves dividing the intersection area between predicted and ground truth regions by their union area, with the mathematical formulation presented as follows:

(Equation 7) $$\begin{array}{c} mIoU=\frac{1}{k+1}\sum_{i=0}^{k}\;\frac{TP}{FN+FP+TP} \end{array}$$

In the field of image segmentation, the Dice coefficient serves as a widely adopted evaluation metric for assessing model performance in image segmentation tasks. It evaluates model accuracy based on the overlap degree between segmentation results. The calculation of the Dice coefficient involves the ratio of the intersection between ground truth and predicted regions to the sum of their areas, with the mathematical formulation expressed as follows:

(Equation 8) $$\begin{array}{c} \text{Dice}=\frac{2\times \text{TP}}{2\times \text{TP}+\text{FP}+\text{FN}} \end{array}$$

The F1 score is a metric used to evaluate the performance of classification models, as it comprehensively considers both Precision (the ratio of correctly predicted positive instances to all instances predicted as positive) and Recall (the ratio of correctly predicted positive instances to all actual positive instances). In binary classification tasks, Precision quantifies the proportion of model-predicted positive samples that are truly positive, while Recall measures the fraction of actual positive cases successfully identified by the model. The F1 score can be calculated using the following formula:

(Equation 9) $$\begin{array}{c} F1=2\times \frac{TP}{2\times TP+FP+FN} \end{array}$$

The Accuracy (ACC) metric evaluates the performance of classification models by measuring the proportion of correctly predicted samples out of the total number of samples. It is calculated using the following formula:

(Equation 10) $$\begin{array}{c} ACC=\frac{TP+TN}{TP+TN+FP+FN} \end{array}$$

#### Statistical analysis

A paired *t* test was performed on the mIoU values of 50 paired test samples (UNet vs. HFSUNet) to validate HFSUNet’s performance improvement, using Python 3.8 (SciPy 1.7.3 for *t* test, numpy 1.21.6 for descriptive statistics).

Sample size: *n* = 50 (50 independent paired test samples).

Data representation: Results are reported as mean, median, SD, SE, Min, and Max (Table 6).

Significance test: A one-tailed paired *t* test was used with α = 0.05.

HFSUNet achieved a mean mIoU of 99.7350% (0.0497% higher than UNet’s 99.6853%), with a median difference of 0.0530% (SD = 0.0766%, range: −0.1091%–0.2067%). The paired *t* test yielded t = 4.5876 (df = 49), one-tailed *p* = 1.6 × 10−5 (*p* < 0.001), and Cohen’s d = 0.6488, confirming statistically extreme significance and practical relevance of the improvement.
